# Gene–disease association study of tumor necrosis factor‐α G‐308A gene polymorphism with risk of major depressive disorder: A systematic review and meta‐analysis

**DOI:** 10.1002/brb3.1628

**Published:** 2020-04-19

**Authors:** Xin Wang, Hongxiu Zhang, Xianling Cao, Wei Shi, Xiaoyu Zhou, Qian Chen, Ke Ma

**Affiliations:** ^1^ College of Traditional Chinese Medicine Shandong University of Traditional Chinese Medicine Jinan PR China; ^2^ Institute of Virology Jinan Municipal Center for Disease Control and Prevention Jinan PR China; ^3^ Department of Oncology Shandong University of Traditional Chinese Medicine Hospital Jinan PR China

**Keywords:** depression, genetic susceptibility, meta‐analysis, polymorphism, tumor necrosis factor‐α

## Abstract

**Introduction:**

The single nucleotide polymorphism (SNP) of the tumor necrosis factor‐α (TNF‐α) G‐308A gene is the most studied regarding susceptibility to major depressive disorder (MDD). However, results have been controversial perhaps due to the heterogeneous genetic backgrounds influenced by race as well as subtypes of depression.

**Methods and materials:**

A systematic MEDLINE search was performed to retrieve all published studies that identified the connection between the TNF‐α G‐308A gene polymorphism and the risk of MDD.

**Results:**

There was no statistical difference between the allele frequencies or genotypes of TNF‐α G‐308A gene and the depressive patients or healthy subjects in the five models tested. Further, subgroup analyses showed that the TNF‐α G‐308A gene polymorphism also did not confer susceptibility to poststroke, late‐life, maternal, or major depression. Publication bias analysis showed *p* values were more than .05, suggesting that the 9 articles included in the current analysis had no publication bias.

**Conclusion:**

Neither the allele frequencies nor genotypes of TNF‐α G‐308A gene could be served as an independent risk factor of depression.

## Graphical abstract

There was no statistical difference between the allele frequency or genotype of TNF‐α G‐308A gene and the depressive patients or healthy subjects in the five models tested. Further, subgroup analyses showed that the TNF‐αG‐308A gene polymorphism also did not confer susceptibility to post‐stroke, late‐life, maternal or major depression.

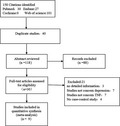

## 1 INTRODUCTION

MDD is a common and debilitating mood disease in the world characterized by pervasive and persistent low mood. It is accompanied by psychological and physiological symptoms, such as lack of motivation, changes in appetite, inability to feel pleasure, cognitive difficulties, and retraction from social interaction (Chan, Cathomas, & Russo, 2019). The proportion of the global population living with MDD is estimated to be 4.4%, representing the primary cause of disability worldwide (Zhao, Li, Tian, Zhu, & Zhao, 2019). Although extensive studies have been investigated over the last few decades, the etiology of depression is still not completely explained, which leads to unclear diagnosis and ineffective pharmacotherapy. This may be attributed to insufficient understanding of the pathogenesis of depression (Wu et al., 2016).

The current consensuses are that depression may be a multifactorial disease with overlapping causal pathways, originating from the environment–gene interaction (Cai et al., 2015). For example, the sustained stressors to the genetically susceptible individuals lead to the deficits of neuron/glial cells, monoamine, neurotrophic factors, and cytokines, subsequently resulting in the increased risk of the onset or relapse of MDD (Dong et al., 2016; Huang et al., 2018). Therefore, numerous hypotheses have been proposed to elucidate its occurrence. Among them, inflammation hypothesis has long been considered as the important pathological mechanism of depression. The evidences from clinical studies have widely demonstrated aberrant inflammation profiles of MDD patients in either central neural system (CNS) or peripheral tissues (Zunszain, Hepgul, & Pariante, 2013). Additionally, proinflammatory cytokines have shown the influence of pathogenesis of depression and antidepressant therapies, such as neurotransmitter metabolism, neuroendocrine function, and regional brain activity (Carlier et al., 2019; Guo et al., 2019; Leonard & Wegener, 2019). Therefore, chronic low‐grade activation of inflammation and the immune system is likely to be served as a risk factor for the occurrence of depression (Mattina, Van Lieshout, & Steiner, 2019; Sonsin‐Diaz et al., 2019; Yin et al., 2019).

TNF‐α is a cytokine involved in the systematic inflammation and regulated immune cells. Recent meta‐analyses confirmed that the concentration of TNF‐α in depressed patients was considerably higher than that of healthy control (Dowlati et al., 2010). Accumulated evidence of clinical trials suggested that decreased level of TNF‐α in blood was related to the improvement of depressive symptoms, and additionally, normalizing the blood concentration of TNF‐α with antidepressants or electroconvulsive therapy could successfully cure depression (Howren, Lamkin, & Suls, 2009). Moreover, inhibition of TNF‐α synthesis through drug blockers can reverse the depressive symptoms of patients (Hannestad, DellaGioia, & Bloch, 2011). Thus, these evidences identified that TNF‐α was likely to be a potential peripheral biomarker for MDD.

Recently, there is an increasing trend for predicting phenotypes associated with susceptible behavioral or biological factors by functional polymorphisms of regulatory genes in the promoter region. As such, it is credible to speculate that identifying susceptibility genes may eventually lead to targeted “cure therapeutics,” giving impetus to identify underlying susceptibility genes associated with MDD. The gene encoding TNF‐α is located on the chromosome 6, which has been reported to be a genetic MDD‐susceptibility region. There are polygene polymorphism loci in TNF‐α gene region (Ma, Zhang, & Baloch, 2016). In the promoter region of the TNF‐α gene, nucleotide −308 with G to A substitution polymorphism affecting its own genetic transcriptional activity was the most studied. However, the controversial results were obtained from the heterogeneous genetic background that influences by race‐related differences as well as the type of depression.

Thus, we conducted a systematic review and meta‐analysis, including case–control studies comparing TNF‐α G‐308A in patients with depressive disorders versus controls without psychopathology. In this paper, we lay out the results of our meta‐analysis and frame them within the context of the current human literature on TNF‐α G‐308A gene polymorphism and depression susceptibility, providing evidence for the role of TNF‐α G‐308A gene polymorphism in depression and identifying gaps in the literature to inform future research.

## 2 METHODS AND MATERIALS

### 2.1 Identification of eligible studies

Three independent researchers conducted a systematic search in PubMed, Cochrane, Web of Science, and EMBASE databases, and the final search update was on 15 May 2019. The following terms were used: “mood disorders” or “depressive disorder” or “depressive episode” or “depression” and “genetic polymorphism” or “genetic variation” or “genetic variant” or “polymorphism” or “single nucleotide polymorphism” or “variant” and “tumor necrosis factor” or “TNF‐alpha.” The language was limited to English. The reference lists of retrieved reports and recent reviews were also manually searched for any additional related articles.

### 2.2 Inclusion and exclusion criteria

The included studies must satisfy the following criteria: (a) The study utilized a case–control design, and all cases must be clearly diagnosed as depression as well as all controls must not with mental illness; (b) the study examined the associations between TNF‐α G‐308A gene polymorphism and depressive disorders; (c) the data are allowed to be calculated by ORs (odds ratios) and 95% CIs (confidence intervals); and (d) the articles were limited to English language. Research that does not meet the inclusion criteria will be excluded. The selection of studies was completed by two independent investigators (Wang and Cao), through reviewing titles, abstracts, and full text. When disagreement arose, it was decided by all researchers.

### 2.3 Data extraction and quality assessment

Data from selected studies were extracted independently by two researchers (Wang and Cao) and then were evaluated in terms of the methodological quality assessment scale. The following details were collected: first author's surname, publication year, type of depression, ethnicity of participants, sizes of case sample and control sample, genotyping methods, diagnostic criteria genotype distribution, HWE score, and NOS score. The methodological quality of the eligible researches was assessed by the NOS. Final scores ranged from 0 to 9 points; scores of 0–5 and 6–9 were deemed to be low and high quality, respectively. Divergences were handled by discussion among all researchers.

### 2.4 Statistics analysis

The software Stata 12.0 and SPSS 21.0 were used to analyze the data. The genotypes’ frequencies of control groups were tested by HWE using the chi‐square test. If *p* > .05, the genotypes’ frequency of control groups was consistent with HWE. The following five genetic comparison models were evaluated: allele genetic model (G vs. A), homozygote genetic model (GG vs. AA), heterozygote genetic model (GA vs. GG), recessive genetic model (AA vs. GA + GG), and dominant genetic model (GG vs. AA + GA). The pooled effect size was estimated by ORs and its 95% CI. The heterogeneity between the included studies was based on chi‐square test and *I* ^2^ statistics. If *I* ^2^ < 50%, it was considered that substantial heterogeneity could be ignored, and then fixed‐effect mode was served to evaluate the pooled ORs and 95% CIs. Apart from this, we performed subgroup analyses by the type of depression. Begg's funnel plot was served to evaluate potential publication bias.

## 3 RESULTS

### 3.1 Literature search

**FIGURE 1:**
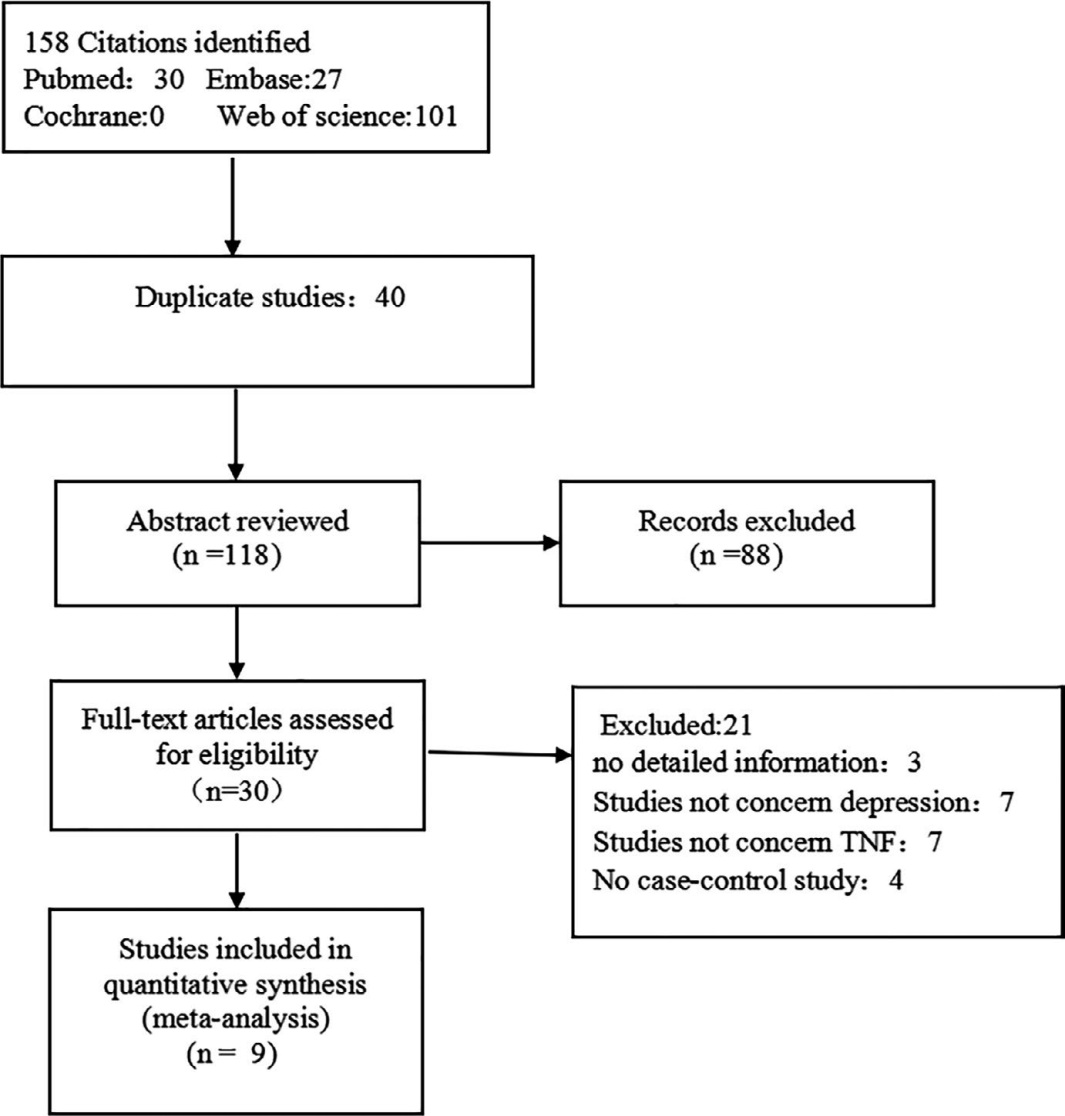
Flowchart of the selection of researches and reason for elimination

The detailed literature search is presented in Figure 1. We removed 40 duplicate records by EndNote 7.0 software. After browsing the titles and abstracts, we excluded 88 studies due to the following reasons: They were reviews, meeting reports, or protocols. Then, 30 studies were potentially relevant, and we further excluded 21 articles due to the listed reasons: The data of cases or controls were incomplete (*n* = 3); they were not gene–disease association research about the role of TNF‐α G‐308A gene polymorphism played in depressive disorders (*n* = 14); or they were not randomized controlled trial (*n* = 3). Eventually, 9 reports satisfied the criteria were included in this meta‐analysis (Cerri et al., 2010; Clerici et al., 2009; Jun et al., 2003; Kang et al., 2015; Kim et al., 2012, 2013, 2017; Mihailova et al., 2016; Sandoval‐Carrillo et al., 2018). Our study does not require ethical approval.

### 3.2 Characteristics of the studies

Table 1 summarizes several main factors of the articles selected in this meta‐analysis. These reports were published from 2003 to 2018, consisting of 717 cases and 2,414 controls. Geographically, one study assessed the Caucasian population and eight assessed the Asian population. The frequencies of genotypes in controls were fully in conformity with HWE. We included nine case–control studies as different types of depression, five as major depression, two as postdepression, one as late‐life depression, and one as maternal depression. NOS scores in this meta‐analysis ranged from 5 to 7. The cases were diagnosed using DSM‐IV, GDS, MMSE, GMS B3, and ICD‐10 scales, and 9 studies applied real‐time PCR as the genotyping method.

**TABLE 1:** Main characteristics of the included studies in the meta‐analysis

Study	Year	Disease	Ethnicity	Sample (Case/Control)	Genotyping	Diagnostic criteria	Cases			Controls			HWE (Controls)	NOS
							GG	GA	AA	GG	GA	AA		
Jun et al	2003	Major depression	Asian	108/125	PCR	DSM‐IV	79	26	3	107	18	0	*p* = .598	6
Cerri et al.	2010	Major depression	Asian	50/240	PCR‐SSP	DSM‐IV	42	8	0	164	65	11	*p* = .682	6
						GDS≧15								
						MMSE≦24								
Clerici et al.	2009	Major depression	Asian	32/363	PCR‐SSP	DSM‐IV Physical examination	23	7	1	277	78	5	*p* = .997	6
Kim et al.	2012	Poststroke depression	Asian	77/199	PCR	DSM‐IV	63	14	0	166	33	0	*p* = .565	5
Kim et al.	2013	Late‐life depression	Asian	63/458	PCR	GMS B3	50	12	1	373	76	9	*p* = .457	7
Kang et al.	2015	Major depression	Asian	101/631	/	GMS B3	81	17	3	507	114	10	*p* = .707	7
Mihailova et al.	2016	Major depression	Caucasian	80/52	PCR‐SSP	ICD‐10	43	9	0	67	13	0	*p* = .594	5
Kim et al.	2017	Poststroke depression	Asian	53/169	PCR	DSM‐IV	31	18	4	121	42	6	*p* = .772	6
Sandoval‐Carrillo et al.	2018	Maternal depression	Asian	153/177	PCR	/	136	17	0	160	14	3	*p* = .413	6

### 3.3 Meta‐analysis of TNF‐α G‐308A gene polymorphism in MDD

**FIGURE 2:**
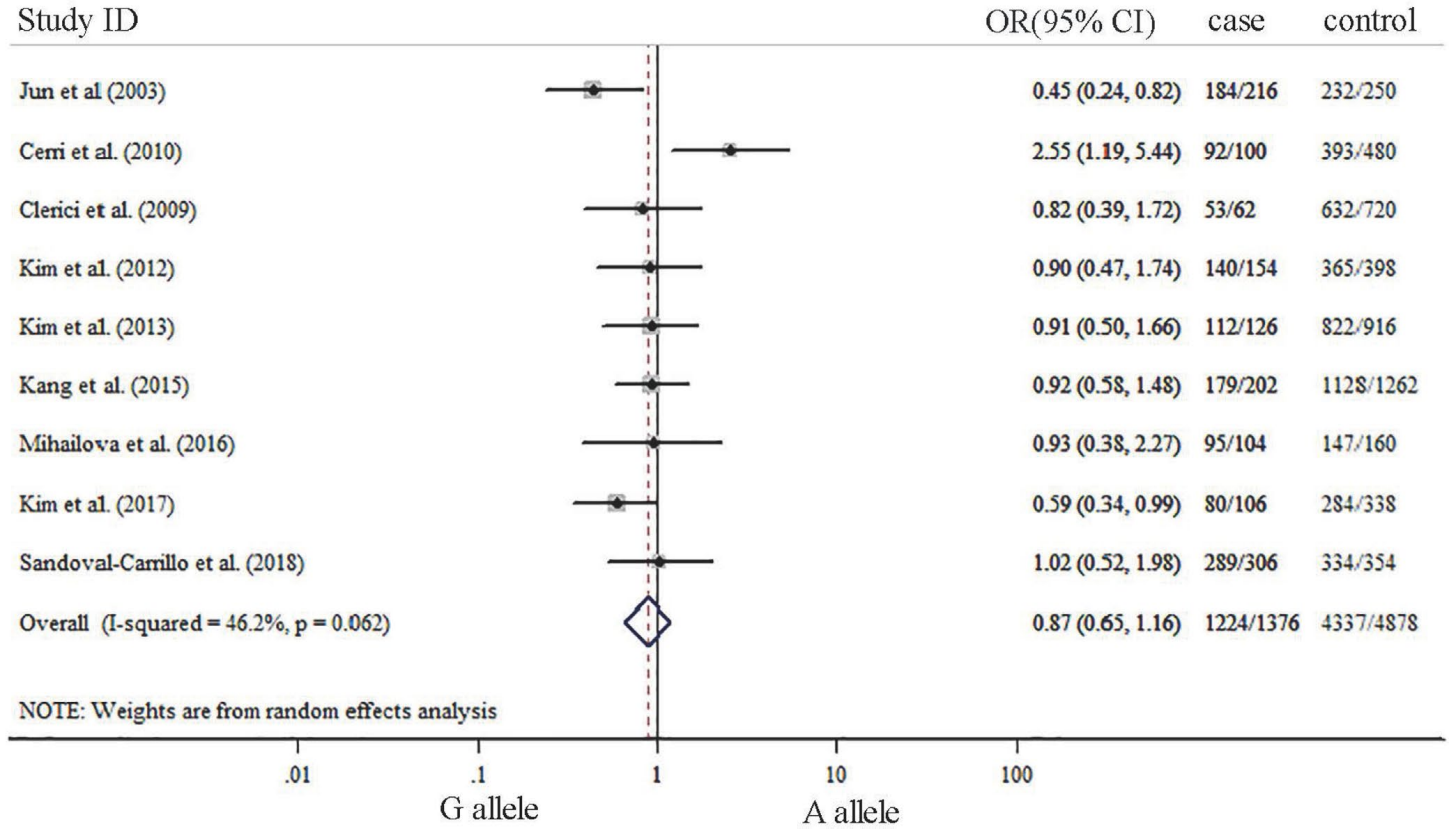
Forest plots of TNF‐α G‐308‐A gene polymorphism in depression: G allele versus A allele

**FIGURE 3:**
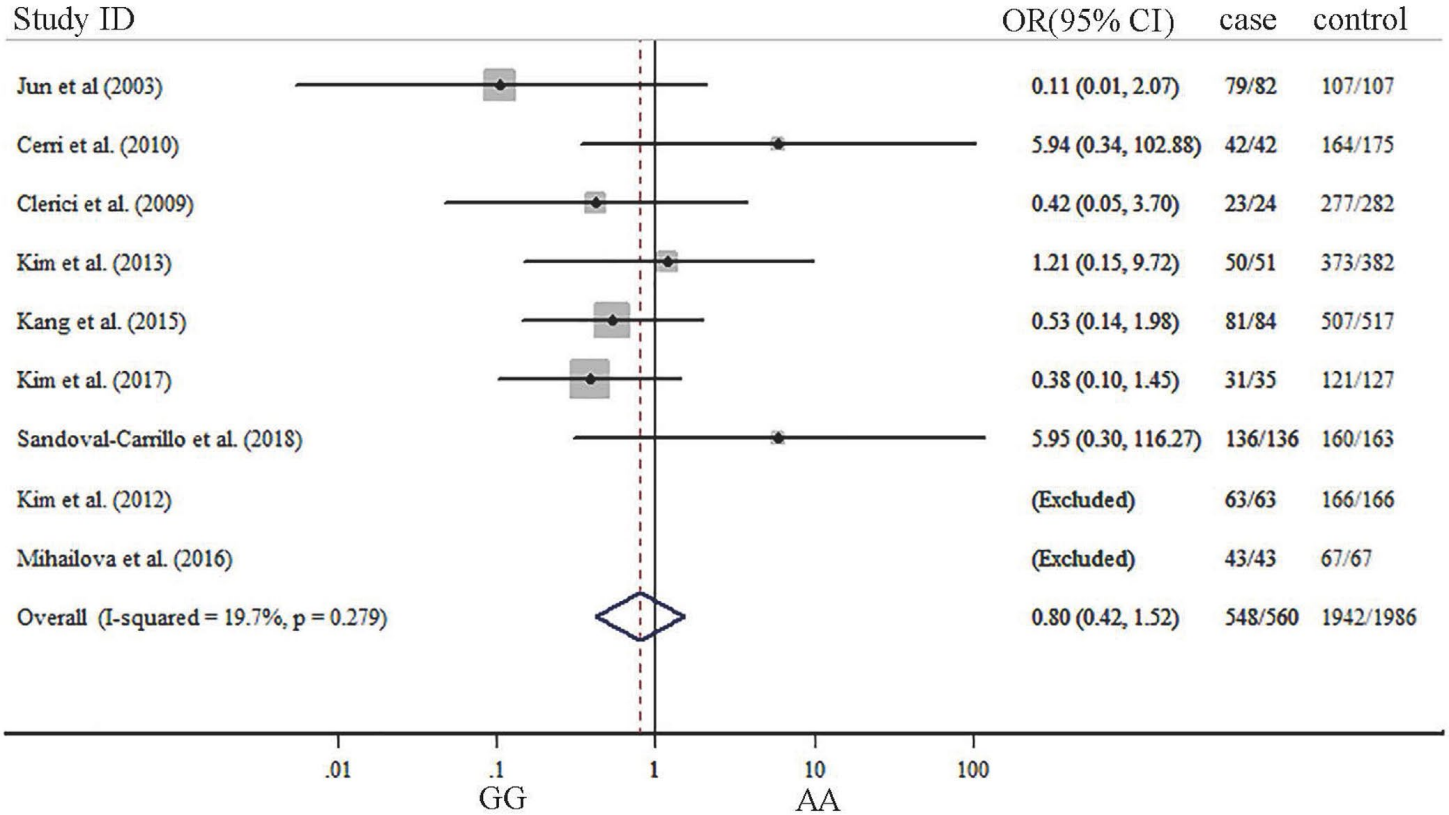
Forest plots of TNF‐α G‐308‐A gene polymorphism in depression: GG versus AA

**FIGURE 4:**
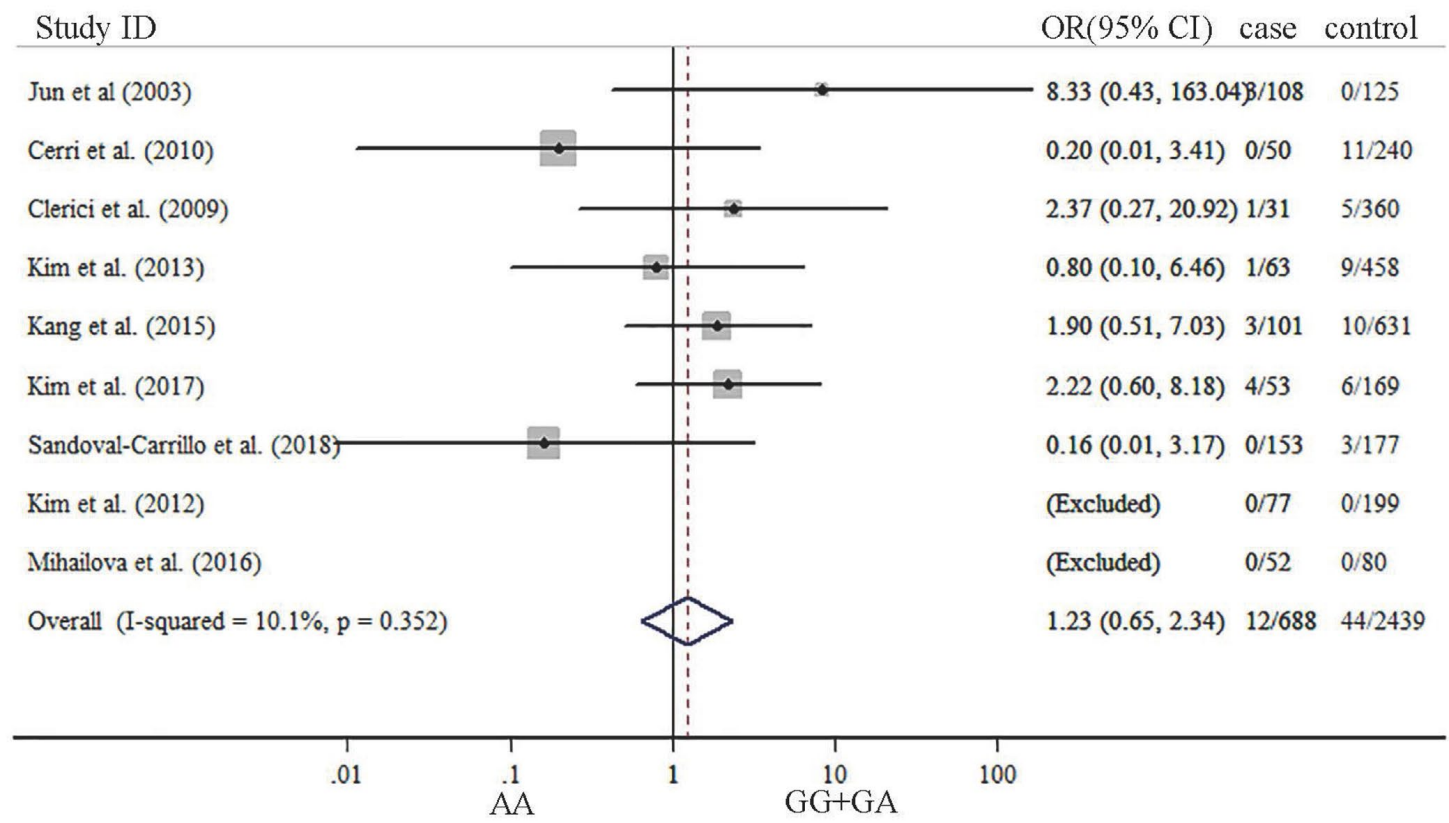
Forest plots of TNF‐α G‐308‐A gene polymorphism in depression: GG versus GA

**FIGURE 5:**
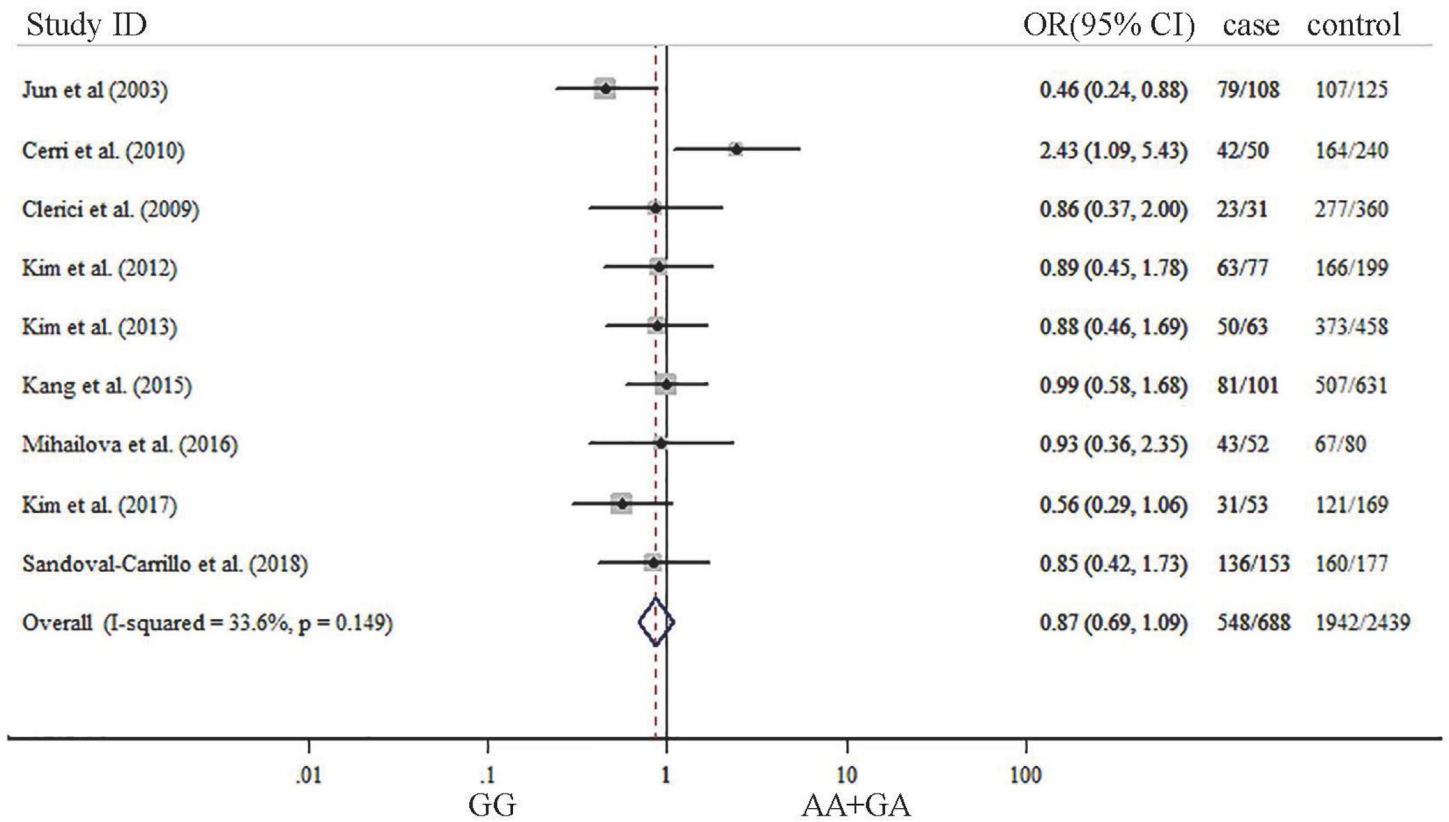
Forest plots of TNF‐α G‐308‐A gene polymorphism in depression: AA versus GG + GA.

**FIGURE 6:**
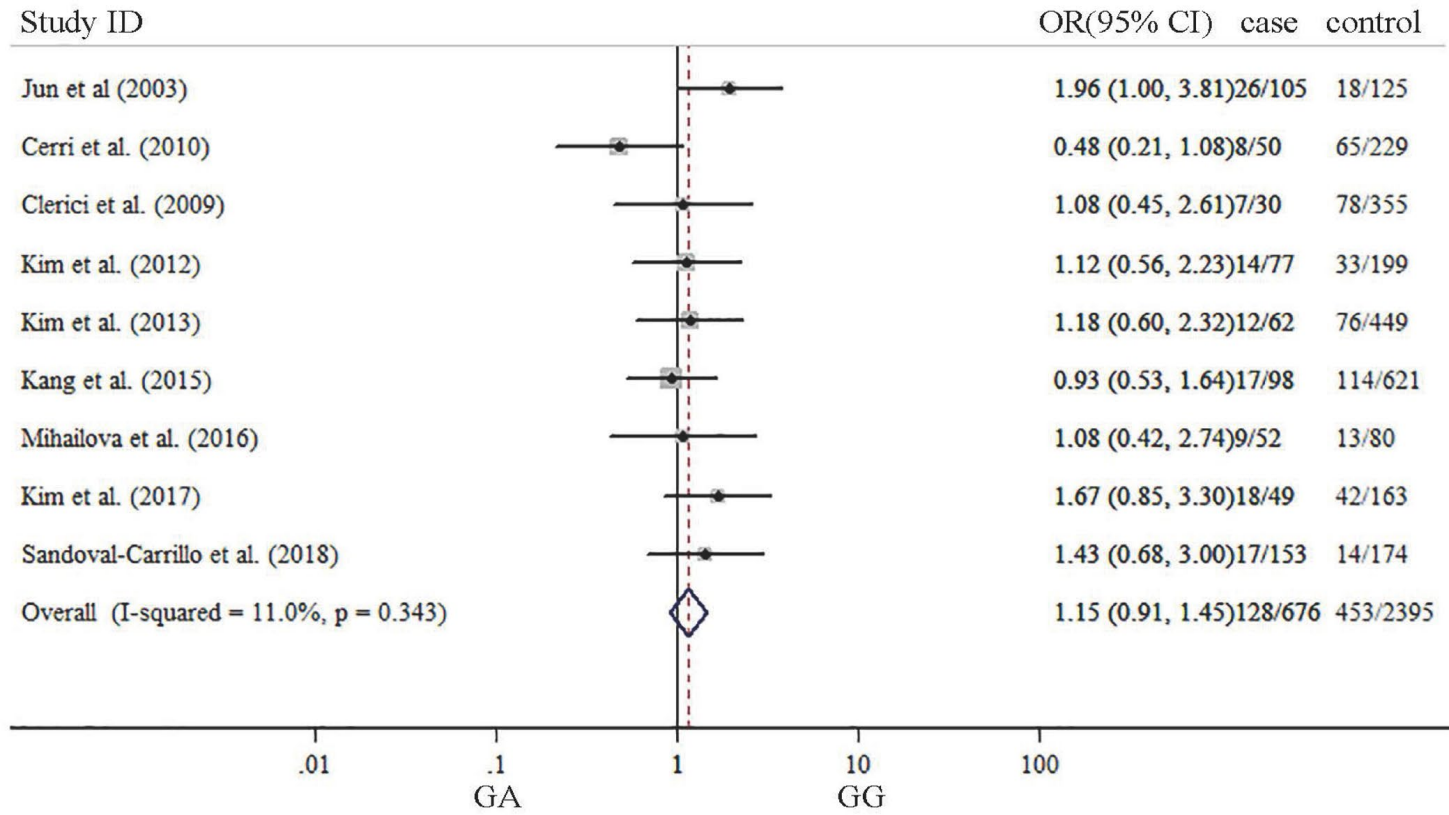
Forest plots of TNF‐α G‐308‐A gene polymorphism in depression: GG versus GA + AA

The summary results about meta‐analysis are listed in Table 2. Our results indicated that significant association was not observed between TNF‐α G‐308A gene polymorphism and depression in five genetic models. ORs with corresponding 95% CIs for the influence of TNF‐a G‐308A gene polymorphism played on depression are detailed in Figures 2, 3, 4, 5, 6. There was no statistically difference under five models (G vs. A: OR: 0.87, 95% CI: 0.65–1.16, *p* = .353, Figure 2; GG vs. AA: OR: 0.80, 95% CI: 0.42–1.52, *p* = .500, Figure 3; GA vs. GG: OR: 0.87, 95% CI: 0.69–1.10, *p* = .343 Figure 4, AA vs. GG + GA: OR: 1.23, 95% CI: 0.65–2.34, *p* = .218, Figure 5; GG vs. GA + AA: OR: 0.87, 95% CI: 0.69–1.09, *p* = .529, Figure 6).

**TABLE 2:** Main results in the total analysis

Group	*I* ^2^, *p* value	OR (95% CI)	*p* value
G versus A	46.2%, 0.062	0.87 (0.65, 1.16)	.353
GG versus GA	19.7%, 0.279	0.80 (0.42, 1.52)	.500
GA versus GG	11.0%, 0.343	0.87(0.69, 1.10)	.343
AA versus GG + GA	10.1%, 0.352	1.23 (0.65, 2.34)	.529
GG versus GA + AA	33.6%, 0.149	0.87 (0.69, 1.09)	.218

### 3.4 Meta‐analysis of TNF‐α G‐308A gene polymorphism in the different types of MDD

In order to eliminate the effect of the type of depression, we subsequently performed subgroup analyses for each genetic model. The results of the four subtype depressions are presented in Table 3. For major depression, there was no statistical difference under five genetic models (G vs. A: OR: 0.93, 95% CI: 0.55–1.59; GG vs. AA: OR: 0.72, 95% CI: 0.31–1.67; GA vs. GG: OR: 0.98, 95% CI: 0.72, 1.35; GG vs. GA + AA: OR: 0.95, 95% CI: 0.70–1.29; AA vs. GG + GA: OR: 1.45, 95% CI: 0.62, 3.43).

**TABLE 3:** Main results in the subgroup analysis

Type of depression	Number	OR (95% CI) G versus A	OR (95% CI) GG versus AA	OR (95% CI) GA versus GG	OR (95% CI) GG versus GA + AA	OR (95% CI) AA versus GA + GG
Major depression	5	0.93 (0.55–1.59)	0.72 (0.31–1.67)	0.98 (0.72–1.35)	0.95 (0.70–1.29)	1.45 (0.62–3.43)
Poststroke depression	2	0.70 (0.46–1.06)	0.38 (0.10–1.45)	0.73 (0.45–1.18)	0.70 ( 0.44–1.11)	2.22 (0.60–8.18)
Late‐life depression	1	0.92 (0.51–1.67)	1.21 (0.15–9.72)	0.85 (0.43–1.67)	0.88 (0.46–1.69)	0.82 (0.10–6.46)
Maternal depression	1	1.02 (0.52–1.98)	5.95 (0.305–116.27)	0.70 (0.33–1.47)	0.85 (0.42–1.73)	0.16 (0.01–3.17)

For poststroke depression, there was no statistical difference under five genetic models: (G vs. A: OR: 0.70, 95% CI: 0.46–1.06; GG vs. AA: OR: 0.38, 95% CI: 0.10–1.45; GA vs. GG: OR: 0.73, 95% CI: 0.45–1.18; GG vs. GA + AA: OR: 0.70, 95% CI: 0.44– 1.11; AA vs. GG + GA: OR: 2.22, 95% CI: 0.60– 8.18).

For late‐life depression, there was no statistical difference under five genetic models: (G vs. A: OR: 0.92, 95% CI: 0.51–1.67; GG vs. AA: OR: 1.21, 95% CI: 0.15–9.72; GA vs. GG: OR: 0.85, 95% CI: 0.43–1.67; GG vs. GA + AA: OR: 0.88, 95% CI: 0.46–1.69; AA vs. GG + GA: OR: 0.82, 95% CI: 0.10–6.46).

For maternal depression, there was no statistical difference under five genetic models: (G vs. A: OR: 1.02, 95% CI: 0.52–1.98; GG vs. AA: OR: 5.95, 95% CI: 0.305–116.27; GA vs. GG: OR: 0.70, 95% CI: 0.33–1.47; GG vs. GA + AA: OR: 0.85, 95% CI: 0.42–1.73; AA vs. GG + GA: OR: 0.16, 95% CI: 0.01–3.17). Unfortunately, TNF‐α G‐308A gene polymorphism also did not affect the susceptibility of different types of depression.

### 3.5 Bias analysis and sensitivity analysis

**FIGURE 7:**
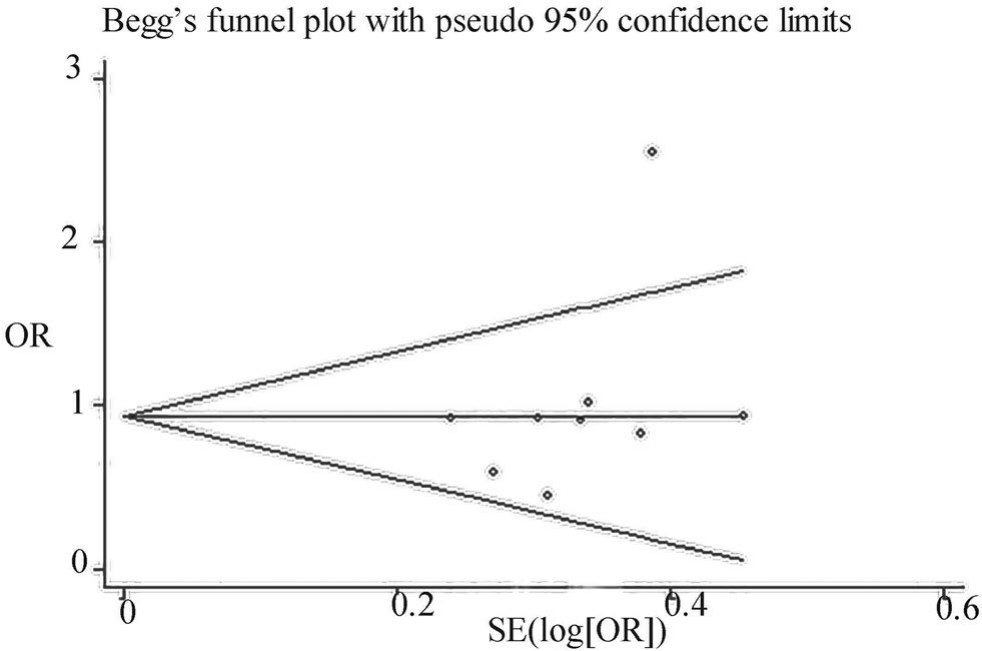
Funnel plots of publication bias

Taking G versus.A as an example, Begg's funnel plot showed that the distribution of each study on both sides of the funnel was basically symmetrical, and Begg's test shows that *p* value was greater than .05 (Figure 7). These results suggested that there was nonsignificant change of publication bias in the current meta‐analysis. Thus, we did not perform the sensitivity analysis.

## 4 DISCUSSION

In the last decade, there is an increasing trend for analyzing functional polymorphisms of regulatory genes in the promoter region to predict phenotypes associated with susceptible behavioral or biological factors (Gupta, Gupta, Bhatia, Tripathi, & Gupta, 2017). Since the imbalance of inflammatory cytokines involved in the pathogenesis of depressive disorders and SNPs in cytokine genes has a bearing on increased secretion or expression of inflammatory biomarkers, there is increasing evidence of investigating the relationship between SNP in inflammation‐related genes and the risk of MDD (Cytokines, 2016).

There are growing evidences from clinical and preclinical studies implying that TNF‐α is potentially to play a crucial role in the pathogenesis of depression. At the molecular level, genome‐wide association study suggested that TNF‐α was genetically associated with MDD (Yao et al., 2015). It has been well established that TNF‐α G‐308A is located in the promoter of TNF‐α and its polymorphism could affect the transcription of TNF‐α production (Richter et al., 2014). The A allele of this polymorphism can lead to high binding affinity of nuclear factors to the TNF promoter, leading to the high level of transcription activity and secretion levels of TNF‐α. So, TNF‐α G‐308A polymorphism was suggested to be a potential risk factor in the depression episode.

So far, comprehensive investigations concerning the association between TNF‐α gene polymorphisms and susceptibility to depressive disorder have been carried out in various races, especially TNF‐α G‐308A, but the divergences remain as a result of the heterogeneous genetic background among human, the different quantities of cases in these studies, and the complex pathogenesis of depression. The evidence from Jun et al. was that A allele and the AA genotype of the TNF‐α G‐308A had a higher risk of susceptibility to depression in the Korean population (Jun et al., 2003). On the opposite side, the evidence from Cerri et al indicated that GG genotype was more likely to increase the risk of developing MDD (Cerri et al., 2010). Besides, Kim et al. demonstrated that GG genotype significantly increased the risk of suicide attempts in MDD (Kim et al., 2012). Interestingly, it was a well‐known fact that GG genotype was the dominant genotype of the normal population (Merino, Zhang, Kaslow, & Aissani, 2013). However, several other studies supported that TNF‐α G‐308A gene polymorphism did not have an effect on depression, such as one contrary opinion was that this SNP was not associated with MDD (Clerici et al., 2009). What's more, Sandoval‐Carrillo et al. first evaluated the possible role TNF‐α G‐308A gene polymorphism played in prenatal depression, and the results showed that there was no correlation between them in the Mexican mestizo population (Kim et al., 2017). Apart from this, higher TNF‐α concentrations were also not associated with TNF‐α G‐308A gene polymorphisms in poststoke depression (Sandoval‐Carrillo et al., 2018).

A meta‐analysis in 2017 had proved that there was no relationship between this SNP of TNF‐α G‐308A gene and depression (Shin, Jeong, Choi, Kim, & Kim, 2017). But, the heterozygote genotype of TNF‐α G‐308A gene was not be analyzed. Moreover, the type of depression may affect the susceptibility to depressive disorder. Since some works suggested that there was an association between this SNP and major depression, others indicated that this SNP did not have a bearing on both prenatal depression and poststoke depression (Sandoval‐Carrillo et al., 2018). Hence, concerning the controversial literature and confusing conclusions, we performed an updated meta‐analysis. We attempted to find the genuine role the allele frequencies and genotypes of this SNP played in depression.

In this study, a total of nine case–control studies encompassing 717 cases and 2,414 controls were included in this meta‐analysis. We performed HWE test on the control groups, and the *p* values of them were all greater than .05. And the NOS scores were all greater than 4 points according to the quality evaluation. It could be considered that the researches were true and reliable. In addition, we also performed Begg's test on the included literature, and the result showed that there was no publication bias. We did not find any evidence that the allele frequencies and genotypes of this SNP were related to depression (G vs. A: OR: 0.87, 95% CI: 0.65–1.16, *p* = .353; GG vs. AA: OR: 0.80, 95% CI: 0.42–1.52, *p* = .500; GA vs. GG: OR: 0.87, 95% CI: 0.69–1.10, *p* = .343; GG vs. GA + AA: OR: 0.87, 95% CI: 0.69–1.09, *p* = .529; AA vs. GG + GA: OR: 1.23, 95 CI: 0.65–2.34, *p* = .218).

We also performed subgroup analyses on the type of depression. However, there were still no statistically significant associations in the subgroup analyses, which could first confirm that the connection between TNF‐α G‐308A gene polymorphisms and MDD was not affected by the type of depression. This discrepancy might be due to the small sample size of this meta‐analysis study, especially the small number of cases. Thus, the sampling error could not be ignored. What's more, it was a well‐known fact that there was a relationship between depression and the increased TNF‐α plasma level. However, evidence suggested that the genetic predisposition to a higher synthesis of this proinflammatory cytokine was associated with a risk of some physical illnesses rather than depression itself (Mihailova et al., 2016). Furthermore, a study of Zhang demonstrated that genotypes and alleles of IFN‐λ genes were not associated with HCV infection, but they had a potential effect on biochemical features of HCV patients (Zhang et al., 2014). Thus, the allele frequencies and genotypes might be connected with the level of TNF‐α instead of the depression.

However, there were still many shortcomings in this meta‐analysis. First, the number of researches we included and the sample sizes of subgroups were limited and the number of cases was less than the controls. Second, depression is influenced by multiple risk factors, such as race, age, sex, and interaction between environment and gene. They all may affect the susceptibility to depressive disorders. The studies we included mainly concerned Asian, elderly and women, which might result in a selective bias. Third, language bias, due to the English limitation in language, some potential researches may be omitted. Nonetheless, this meta‐analysis provides an evaluation about the potential effect that the TNF‐α G‐308A gene polymorphism has on depression.

## 5 CONCLUSION

In summary, neither the allele frequencies nor genotypes of TNF‐α G‐308A gene could be served as an independent risk factor of depression. What's more, this relationship was also not related to the type of depression. Considering that the incidence of depression is polygenic and it relates to many factors, such as race, environment, gender, and age, the connection between the SNP of TNF‐α G‐308A gene and depression is still required to be set forth by large sample case–control studies.

## CONFLICT OF INTEREST

All authors declare no competing interest. All funding sources supported only the execution of the experiments. We strictly disclosed that all funders had no role in the study design, data collection and analysis, decision to publish, or preparation of the manuscript.

## AUTHORS CONTRIBUTIONS

X Wang, HX Zhang, XY Zhou, Q Chen, and XL Cao contributed to literature search, data extraction, and data analysis. K MA and W Shi contributed to the project design and paper writing.

## ETHICAL STATEMENT

Our study does not require ethical approval.

## Data Availability

The raw data supporting the conclusions of this manuscript will be made available by the authors, without undue reservation, to any qualified researcher.
